# Is There a Role for Glutaredoxins and BOLAs in the Perception of the Cellular Iron Status in Plants?

**DOI:** 10.3389/fpls.2019.00712

**Published:** 2019-06-04

**Authors:** Pascal Rey, Maël Taupin-Broggini, Jérémy Couturier, Florence Vignols, Nicolas Rouhier

**Affiliations:** ^1^Plant Protective Proteins Team, CEA, CNRS, BIAM, Aix-Marseille University, Saint-Paul-lez-Durance, France; ^2^Biochimie et Physiologie Moléculaire des Plantes, CNRS/INRA/Université de Montpellier/SupAgro, Montpellier, France; ^3^Université de Lorraine, INRA, IAM, Nancy, France

**Keywords:** BOLA, glutaredoxins, iron–sulfur center, maturation factor, iron homeostasis

## Abstract

Glutaredoxins (GRXs) have at least three major identified functions. In apoforms, they exhibit oxidoreductase activity controlling notably protein glutathionylation/deglutathionylation. In holoforms, i.e., iron–sulfur (Fe–S) cluster-bridging forms, they act as maturation factors for the biogenesis of Fe–S proteins or as regulators of iron homeostasis contributing directly or indirectly to the sensing of cellular iron status and/or distribution. The latter functions seem intimately connected with the capacity of specific GRXs to form [2Fe–2S] cluster-bridging homodimeric or heterodimeric complexes with BOLA proteins. In yeast species, both proteins modulate the localization and/or activity of transcription factors regulating genes coding for proteins involved in iron uptake and intracellular sequestration in response notably to iron deficiency. Whereas vertebrate GRX and BOLA isoforms may display similar functions, the involved partner proteins are different. We perform here a critical evaluation of the results supporting the implication of both protein families in similar signaling pathways in plants and provide ideas and experimental strategies to delineate further their functions.

## Introduction

Many cellular reactions and biological processes require metalloproteins, among which those containing iron (Fe) cofactors such as mononuclear and dinuclear (non-heme) Fe centers, hemes and iron–sulfur (Fe–S) clusters, are particularly crucial. Unlike other metals such as copper or zinc, there is no universal Fe chaperone described and so far, only poly rC-binding proteins (PCBPs) were shown to coordinate Fe entry in the cytosol and serve for the metalation of non-heme Fe enzymes in mammals (Philpott et al., 2017). In contrast, the synthesis/assembly of hemes and Fe–S clusters requires more complex and universally conserved pathways (Couturier et al., 2013; Barupala et al., 2016). The machineries dedicated to the maturation of Fe–S proteins present in mitochondria and chloroplasts, named ISC (iron–sulfur cluster) and SUF (sulfur mobilization), respectively, are also found in bacteria (Lill, 2009). On the other hand, cytosolic and nuclear Fe–S proteins are maturated via the eukaryote-specific cytosolic iron–sulfur cluster assembly (CIA) machinery, which is, however, dependent on the mitochondrial ISC machinery for sulfur supply (Lill, 2009). Hence, given the high cellular demand for iron, sophisticated systems exist to control Fe uptake and intracellular distribution due to its potential toxicity. Strikingly, the Fe sensing systems and associated transcription factors generally differ in bacteria, yeast/fungi, mammals, and plants, but might include common actors such as glutaredoxins (GRXs) and BOLAs (Couturier et al., 2015).

Two GRX classes, I and II, are present in most organisms whereas additional classes are specific to some species/genus/kingdoms (Alves et al., 2009; Couturier et al., 2009). GRXs of the first class are involved in redox regulation, reducing protein disulfides or glutathione-protein mixed disulfides. GRXs from class II participate in the regulation of Fe homeostasis (Mühlenhoff et al., 2010; Haunhorst et al., 2013) and in the maturation of Fe–S proteins owing to their capacity to ligate and exchange [2Fe–2S] clusters with partner proteins (Table 1; Rodríguez-Manzaneque et al., 2002; Bandyopadhyay et al., 2008). They are also referred to as monothiol GRXs or CGFS GRXs owing to their conserved CGFS active site signature.

**Table 1:** Iron-related phenotypes of *bolA* and *glutaredoxin* mutants from various sources.

	Organism	Protein names	Mutant phenotype(s)	References
Mono-domain (organellar) GRXs	*Saccharomyces cerevisiae*	Grx5	Defaults in Fe–S cluster assembly	Rodríguez-Manzaneque et al., 2002; Mühlenhoff et al., 2003
	*Schizosaccharomyces pombe*	Grx5	Defaults in Fe–S cluster assembly, decreased amount of mitochondrial DNA, reduced growth, and sensitivity toward oxidants	Chung et al., 2005; Kim et al., 2010
	*Danio rerio*	GRX5	Embryo lethal	Wingert et al., 2005
	*Homo sapiens*	GLRX5	Defaults in Fe–S cluster assembly leading to sideroblastic anemia	Camaschella et al., 2007; Ye et al., 2010
	*Trypanosoma brucei*	1-C-Grx1	Lethal	Comini et al., 2008
	*Sinorhizobium meliloti*	Grx2	Defaults in Fe–S cluster assembly, deregulation of RirA transcriptional activity, increased intracellular iron content, modified nodule development	Benyamina et al., 2013
	*Escherichia coli*	Grx4	Sensitivity to iron depletion, defect in respiratory complex I	Yeung et al., 2011; Burschel et al., 2019
	*Arabidopsis thaliana*	GRXS14	Sensitivity to prolonged darkness	Rey et al., 2017
	*Arabidopsis thaliana*	GRXS15	Lethal, decreased amounts of lipoate synthase and of lipoic acid dependent H subunits of the glycine cleavage system in RNAi lines	Moseler et al., 2015; Ströher et al., 2016
	*Arabidopsis thaliana*	GRXS16	None described for co-suppressed and RNAi lines	Rey et al., 2017
Multi-domain (cytosolic) GRXs	*Saccharomyces cerevisiae*	Grx3	Impaired regulation of Aft1/2 and iron homeostasis	Ojeda et al., 2006; Pujol-Carrion et al., 2006
	*Saccharomyces cerevisiae*	Grx4	Impaired regulation of Aft1/2 and iron homeostasis	Ojeda et al., 2006; Pujol-Carrion et al., 2006
	*Saccharomyces cerevisiae*	Grx3–Grx4	Lethal in some background. Impaired iron trafficking and assembly of Fe–S proteins, heme, and iron-containing proteins	Pujol-Carrion et al., 2006; Mühlenhoff et al., 2010
	*Schizosaccharomyces pombe*	Grx4	Lethal	Chung et al., 2005
	*Cryptococcus neoformans*	Grx4	Slow growth upon iron deprivation	Attarian et al., 2018
	*Danio rerio*	GRX3	Impaired heme synthesis and Fe–S protein maturation	Haunhorst et al., 2013
	*Homo sapiens*	GLRX3/PICOT	Decreased activities of cytosolic Fe–S proteins	Haunhorst et al., 2013
	*Arabidopsis thaliana*	GRXS17	Growth defects (meristem arrest) upon elevated temperature and long photoperiod. No decrease in cytosolic Fe–S protein activity	Cheng et al., 2011; Knuesting et al., 2015; Yu et al., 2017
BOLA	*Saccharomyces cerevisiae*	Bol1	No growth defect and no decrease in Fe–S enzyme activity	Melber et al., 2016; Uzarska et al., 2016
	*Saccharomyces cerevisiae*	Bol3	Slightly decreased complex II (SDH) activity	Melber et al., 2016; Uzarska et al., 2016
	*Saccharomyces cerevisiae*	Bol1–Bol3	Decreased activity of lipoic acid-dependent enzymes, aconitase, and respiratory complex II	Melber et al., 2016; Uzarska et al., 2016
	*Saccharomyces cerevisiae*	Bol2/Fra2	Impaired regulation of Aft1/2 and iron homeostasis	Kumánovics et al., 2008; Uzarska et al., 2016
	*Schizosaccharomyces pombe*	BolA2/ Fra2	Impaired regulation of the Fep1 transcription factor	Jacques et al., 2014
	*Homo sapiens*	BOLA1	Oxidation of the mitochondrial GSH pool	Willems et al., 2013
	*Homo sapiens*	BOLA2	None described for siRNA lines	Frey et al., 2016
	*Homo sapiens*	BOLA3	Defect in lipoic acid-dependent enzymes and in respiratory complexes I and II	Cameron et al., 2011
	*Escherichia coli*	BolA	Partial defect in respiratory complex I assembly	Burschel et al., 2019
	*Escherichia coli*	IbaG	None described	Burschel et al., 2019
	*Escherichia coli*	BolA – IbaG	Decreased complex II activity	Burschel et al., 2019
	*Salmonella typhimurium*	BolA	Decreased resistance to acidic and oxidative stresses and decreased virulence	Mil-Homens et al., 2018
	*Arabidopsis thaliana*	BOLA2	None described under control conditions, increased resistance to oxidative conditions	Qin et al., 2015


Regarding the BOLA family, an extensive phylogenetic analysis allowed delineating four groups, namely BOLA1–BOLA4 (Couturier et al., 2014). BOLA1s are present in both bacteria and eukaryotes, BOLA2s and BOLA3s in eukaryotes and BOLA4s in photosynthetic organisms, archaea, and bacteria. Pioneer works revealed functions for *Escherichia coli* BolA in the regulation of cell morphology, possibly as a transcriptional regulator (Aldea et al., 1989), for *Saccharomyces cerevisiae* cytosolic Bol2/Fra2 (Fe repressor of activation 2) in the regulation of iron homeostasis (Lesuisse et al., 2005; Kumánovics et al., 2008), and for mitochondrial BOLAs (human BOLA3 and yeast Bol1, Bol3) in the maturation of Fe–S clusters (Table 1; Cameron et al., 2011; Melber et al., 2016; Uzarska et al., 2016).

**FIGURE 1:**
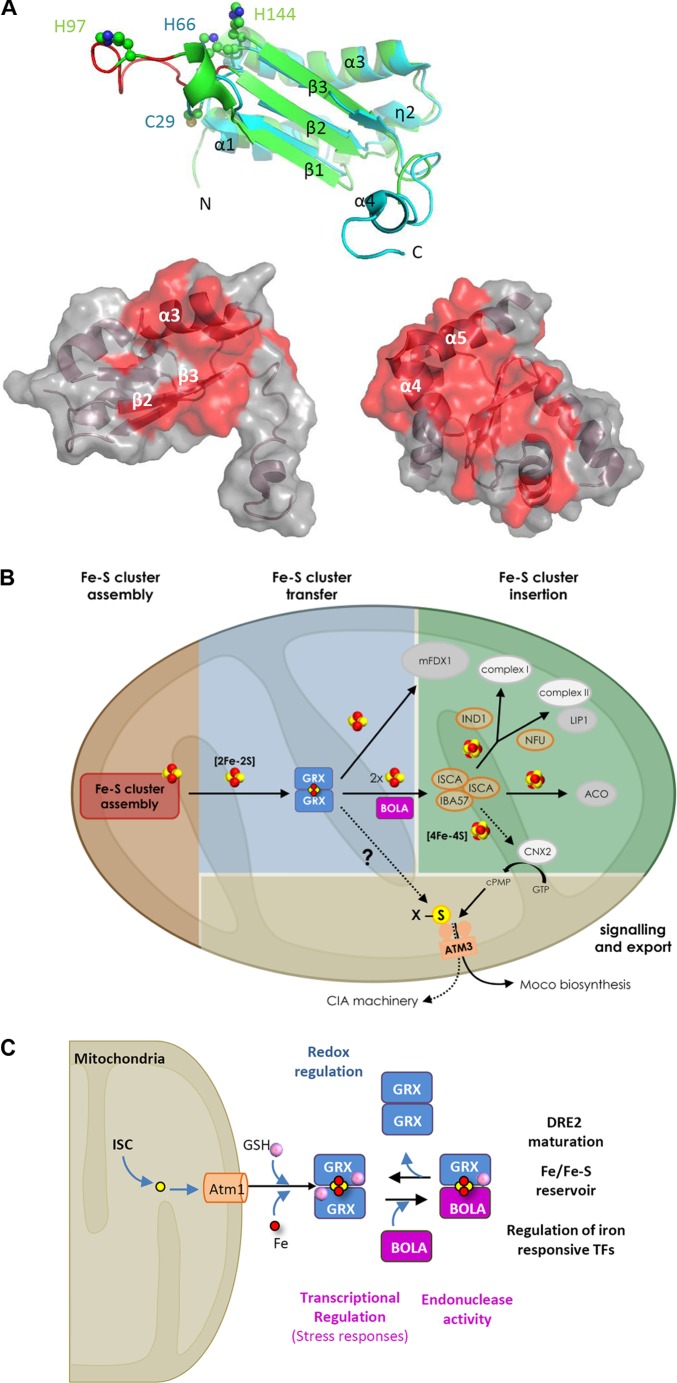
Properties and hypothetical roles of the class II GRX-BOLA couple in plants. **(A)** Tridimensional structures of plastidial *Arabidopsis thaliana* BOLA1 and GRXS14 proteins highlighting the residues involved in the interactions. On the top, superimposition of AtBOLA1 (green) and AtBOLA2 (blue) structures. Both proteins have a α/β-structure made of four helices and three strands with an α1β1β2η2α3β3α4 (η: 3_10_-helix) topology (Roret et al., 2014). The β-strands form a central three-stranded β-sheet. In addition to the extended C-terminal part in AtBOLA2, both proteins differ by the length of the β1–β2 loop (in red in BOLA1), referred to as [H/C] loop, and which contains the histidine (His97 in AtBOLA1) or cysteine (Cys29 in AtBOLA2) residues provided by BOLA proteins for Fe–S cluster bridging together with the His66 (AtBOLA2) or His144 (AtBOLA1). The putative DNA binding site in BOLAs is formed by the η2 and α3 helices, the loop containing a specific FXGX signature (type II β-turn), the α3 helix containing a positively charged RHR motif and the β3 strand. Below, from left to right, AtBOLA2 structure showing 18 residues (mainly part of the β2 and β3 strands and α3 helix) identified by NMR titration as involved in the interaction with apo-AtGRXS14; and AtGRXS14 structure showing 32 residues (many present in the C-terminal α3 and α4 helices) identified by NMR titration as involved in the interaction with AtBOLA2 (Roret et al., 2014). These residues, plus some additional ones, are also involved in the formation of the [2Fe–2S] cluster-bridged heterodimer as determined using human proteins (Nasta et al., 2017). **(B)** Hypothetical model for the role of GRXS15 and BOLA4 in plant mitochondria. By analogy with the yeast system, GRXS15 (shortened as GRX) should receive a [2Fe–2S] cluster synthesized *de novo* by a multi-protein assembly complex (details about the proteins involved in the early steps of Fe–S cluster assembly and transfer have been omitted). GRXS15 is supposed to transfer its [2Fe–2S] cluster to client proteins as the mitochondrial ferredoxin 1 (mFDX1) (Moseler et al., 2015) or to ISCA proteins for the reductive conversion of two [2Fe–2S] clusters into a [4Fe–4S] cluster (as shown with human proteins) and its subsequent delivery to client proteins bearing such cluster. In the absence of genetic analysis about *bola4* mutants, the contribution of BOLA4 (shortened as BOLA) for the respective roles of GRXS15 is unclear, but the confirmed interaction between both proteins (Couturier et al., 2014) prompted us to include BOLA at this step as yeast Bol1/3 proteins are only required for the maturation of [4Fe–4S] proteins. The specific defects observed for aconitase (ACO) and lipoic-acid dependent proteins in the *GRXS15* mutant lines indicate a direct or indirect role for GRXS15 in the maturation of both lipoate synthase (LIP1) and aconitase. Finally, whether GRXS15 is required for the maturation and activity of cytosolic and nuclear Fe–S proteins by fueling the CIA machinery as shown for yeast Grx5, or by indirectly contributing to the synthesis of molybdenum cofactor, that is present in several cytosolic Fe–S proteins, is unknown. **(C)** Roles associated with the various oligomeric forms involving nucleo-cytosolic GRXs and BOLAs irrespective of the organisms considered. The color code is as follows: in blue, functions associated with apo-dimeric GRX forms, in purple those associated with apo-BOLA and in black those associated with the GRX homodimeric or GRX-BOLA heterodimeric forms bridging a [2Fe–2S] cluster.

A very close relationship between class II GRXs and BOLAs was initially evident from genome (gene co-occurrence and clustering, existence of fusion proteins) and large-scale interactomic analyses in various organisms (reviewed in Przybyla-Toscano et al., 2017). Then, the molecular and structural determinants of the complexes were investigated in detail using mutational, spectroscopic and structural analyses on recombinant proteins. This led to demonstrate that class II GRXs and BOLAs form both apo- and holo-heterodimers bridging a [2Fe–2S] cluster, usually more stable than the [2Fe–2S] cluster-bridging GRX homodimers, and to identify the residues serving as ligands (Li and Outten, 2012; Couturier et al., 2015; Przybyla-Toscano et al., 2017). In GRX-BOLA holo-heterodimers, the [2Fe–2S] cluster is ligated using the GRX conserved cysteine, a cysteine from glutathione (as in GRX holo-homodimers), and, on the BOLA side, using a C-terminally located conserved histidine and an histidine or a cysteine in the β1–β2 loop, referred to as [H/C] loop (Figure 1A; Li et al., 2011, 2012; Roret et al., 2014; Dlouhy et al., 2016; Nasta et al., 2017).

Hereafter, based on the most recent results and known roles in non-photosynthetic organisms, we discuss the putative or confirmed functions of GRX and BOLA, alone or in complex, in photosynthetic organisms.

## The Class II GRX and Bola Couple Proteins Present in Bacteria or in Eukaryote Organelles Are Involved in the Synthesis of Fe–S Clusters

The first evidence about GRX involvement in the biogenesis of Fe–S proteins were obtained from a *S. cerevisiae* mutant for the mitochondrial Grx5 (Table 1; Rodríguez-Manzaneque et al., 2002; Mühlenhoff et al., 2003). Orthologs of this single domain-containing GRX are found in bacteria, archaea and plant plastids. The current view is that Grx5 is required for the maturation of all types of Fe–S clusters in mitochondria, receiving a [2Fe–2S] cluster from ISCU-type scaffold proteins and transferring it to ISCA-type transfer proteins for subsequent maturation of [4Fe–4S] proteins (Figure 1B). Grx5 is also required for the maturation of nucleo-cytosolic Fe–S proteins and the activation of the Aft1 transcription factor, pointing to its key position in *S. cerevisiae* (see below) (Uzarska et al., 2013). Yeast Bol1 and Bol3, which have the capacity to form heterodimers with Grx5, were later shown to be required for a specific set of mitochondrial [4Fe–4S] proteins, without affecting *de novo* synthesis of [2Fe–2S] proteins (Uzarska et al., 2016). So far, human BOLA3, but not BOLA1, has been demonstrated as required for the maturation of specific Fe–S proteins (Table 1; Cameron et al., 2011; Willems et al., 2013). The client proteins are notably the succinate dehydrogenase/complex II and lipoate synthase. Moreover, the fact that *bol1–bol3*Δ mutants are neither affected in the CIA machinery, nor in Aft1 activation, indicates that Grx5 has physiological roles independent of Bol1 and Bol3 (Uzarska et al., 2016). Additional studies suggested that Bol1 indeed acts early in the ISC pathway in concert with Grx5 (possibly only for [4Fe–4S] proteins) whereas Bol3 may preferentially act with NFU1, a late Fe–S cluster transfer protein, to preserve the [4Fe–4S] center found in some specific mitochondrial client proteins, as lipoate synthase, from oxidative damage (Figure 1B; Melber et al., 2016).

Concerning bacteria, the sole Grx isoform (Grx4/D) and both BolAs (BolA and IbaG) from *E. coli* were recently shown as implicated in the maturation of the respiratory complexes I and II, but the effects are only visible when multiple genes are mutated (Burschel et al., 2019). This role in maturating Fe–S proteins is consistent with (i) the synthetic lethality of the *Grx4* gene with genes present in the ISC operon (Butland et al., 2008), (ii) the interaction *in vitro* of Grx4 with the MiaB Fe–S protein (Boutigny et al., 2013), and (iii) the capacity of Grx4-BolA and Grx4-IbaG to form the usual [2Fe–2S] cluster-bridging heterodimers (Yeung et al., 2011; Dlouhy et al., 2016). In *Sinorhizobium meliloti*, deletion of the sole class II GRX also leads to impaired maturation of Fe–S proteins and increased intracellular iron content (Benyamina et al., 2013).

In plants, the corresponding mitochondrial GRX is named GRXS15. Knockout Arabidopsis mutants are lethal due to defective embryo development (Moseler et al., 2015). Plants expressing a mutated GRXS15 form modified for its ability to coordinate an Fe–S cluster exhibit severely reduced growth and impaired aconitase activity (Moseler et al., 2015). Additionally, Arabidopsis GRXS15 down-regulated lines display slowed growth and impaired activity of enzymes dependent on lipoic acid, the synthesis of which is ensured by the Fe–S cluster-containing lipoyl synthase (Ströher et al., 2016). Whether GRXS15 fulfills its function in concert with BOLA4, the sole mitochondrial BOLA, remains to be explored, but their interaction was demonstrated in yeast and *in planta* (Figure 1B; Couturier et al., 2014). Plants also have class II GRXs (GRXS14 and S16) and mono-domain BOLAs (BOLA1, BOLA4) in plastids (Couturier et al., 2013). So far, *in planta* evidence for their implication in the biogenesis of Fe–S proteins are scarce (Table 1). GRXS14-deficient Arabidopsis plants exhibit accelerated chlorophyll loss upon prolonged darkness, a treatment also leading to a decreased abundance of proteins acting in Fe–S cluster metabolism (Rey et al., 2017). Nevertheless, the demonstration that Arabidopsis and/or poplar GRXS14 and GRXS16 interact both with BOLA1 and BOLA4 (Couturier et al., 2014), bind Fe–S clusters alone or in complex (Bandyopadhyay et al., 2008; Dhalleine et al., 2014; Roret et al., 2014) and transfer it to partner proteins (Mapolelo et al., 2013) give strong credence to such a role. Even more importantly, all plant *GRX* and *BOLA* genes complement totally or partially (*GRXS15*) the corresponding yeast *grx5* and *bol1–bol3* mutants, indicating that they possess similar structural and functional determinants (Bandyopadhyay et al., 2008; Moseler et al., 2015; Uzarska et al., 2018).

## Multiple Functions in the Regulation of Iron Homeostasis of the Class II GRX and Bola Couple in the Cytosol/Nucleus of Eukaryotes

Eukaryote cytosolic class II GRXs are multidomain proteins formed by an N-terminal thioredoxin-like domain fused to one to three GRX domains (Couturier et al., 2009). Most organisms have a single GRX of this type and also a single cytosolic BOLA isoform, referred to as BOLA2/Bol2/Fra2. The pioneering studies showing the involvement of class II GRXs and BOLAs in Fe homeostasis have been conducted in *S. cerevisiae* mutants deregulated in *Grx3, Grx4*, and *Bol2/Fra2* genes (Table 1; Lesuisse et al., 2005; Ojeda et al., 2006; Pujol-Carrion et al., 2006). In yeast, the regulation of Fe concentration is achieved at the transcriptional level by low-(Aft1 and Aft2) and high-level (Yap5) sensing transcription factors and at the post-transcriptional level by mRNA-binding proteins (Outten and Albetel, 2013). Both types of transcription factors bind [2Fe–2S] clusters allowing them to perceive the cellular Fe or Fe–S cluster status (Poor et al., 2014; Rietzschel et al., 2015). Whereas *Grx4* expression is regulated by Yap5, it is not documented whether Yap5 localization or activity is controlled by a GRX/BOLA complex. Regarding Aft1/Aft2, their subcellular (nuclear vs. cytosolic) localization is controlled by a Fra2-Grx3/4 inhibitory complex (possibly requiring also the aminopeptidase Fra1) (Kumánovics et al., 2008). The current view is that the presence of an Fe–S cluster in the Fra2-Grx3/4 complex is synonymous of iron-replete conditions and of a correct functioning of the ISC machinery (Figure 1C; Kumánovics et al., 2008). By transferring a cluster to Aft1/2, the GRX-BOLA complex should either retain them in the cytosol or promote their dissociation from DNA if in the nucleus (Ueta et al., 2012; Poor et al., 2014).

Some aspects of Fe homeostasis in other yeasts and fungi are also controlled by GRX and/or BOLA. In *Cryptococcus neoformans*, Fe repletion promotes Grx4 relocation from the nucleus to the cytoplasm allowing the regulation of Cir1, a master regulator of Fe-responsive genes (Attarian et al., 2018). In *Schizosaccharomyces pombe*, Fe metabolism is regulated by two transcriptional repressors, the GATA-type iron sensing Fep1 and the CCAAT-binding factor complex subunit Php4 (Brault et al., 2015). Their localization and/or DNA binding activity are regulated by Grx4 and/or Fra2 (reviewed in Outten and Albetel, 2013; Brault et al., 2015). The binding of a [2Fe–2S] cluster between Grx4 and Php4 may promote Php4 release from the CCAAT-binding complex at the DNA targets and suppress its inhibitory effect on the expression of Fe storage genes (Dlouhy et al., 2017). Unlike Php4, the regulation of which does not involve Fra2, the formation of a [2Fe–2S]-Grx4/Fra2 heterodimeric complex is required for regulating Fep1 activation (Jacques et al., 2014; Encinar del Dedo et al., 2015).

In mammals, the regulation of Fe metabolism and homeostasis is ensured by IRP1/2 and RNA-binding proteins (Rouault and Maio, 2017). Under Fe limitation, both IRPs bind to the so-called Iron Responsive Elements (IREs) in untranslated regions of mRNAs coding for proteins implicated in Fe assimilation and homeostasis (Rouault and Maio, 2017). Doing so, they control either mRNA stabilization or translational blocking. Whereas IRP2 release from IREs is mediated by proteasomal degradation (Guo et al., 1995), IRP1 function may depend on GLRX3/PICOT (but also on mitochondrial GLRX5) as it relies on the binding of an Fe–S cluster. Under Fe sufficiency, IRP1 binds a [4Fe–4S] cluster and acts as an aconitase whereas under Fe limitation the protein turns into an apoform binding to IREs. Consequently, IRP1 requires functional mitochondrial and cytosolic Fe–S cluster assembly machineries. Having two GRX domains, human GLRX3 forms homodimers or heterotrimers with two BOLA2 molecules bridging two [2Fe–2S] clusters (Li et al., 2012; Banci et al., 2015b; Frey et al., 2016). It also binds a [4Fe–4S] cluster and transfers it *in vitro* to an apo-IRP1 (Xia et al., 2015). *GLRX3* silencing in human HELA cells decreases the activity of several cytosolic Fe–S proteins, including IRP1 (Table 1; Haunhorst et al., 2013). In zebrafish, *GLRX3* deletion impairs heme biosynthesis during embryo development (Haunhorst et al., 2013). All of this indicates important functions of vertebrate GLRX3 in Fe metabolism.

In addition to an Fe sensing function, an Fe or Fe–S cluster trafficking function was proposed for yeast Grx3/4 and the human GLRX3-BOLA2 complex to ensure proper assembly of several types of Fe-containing centers. In fact, most multidomain GRXs are able to rescue the Fe–S cluster maturation defects of the yeast *grx5* mutant (Molina et al., 2004; Bandyopadhyay et al., 2008; Knuesting et al., 2015) suggesting that they have the capacity of exchanging Fe–S clusters. Accordingly, both human GLRX3 homodimers and GLRX3-BOLA2 trimeric complexes bridging two [2Fe–2S] clusters can deliver their clusters to the anamorsin/CIAPIN/DRE2 protein (Banci et al., 2015a,b). From the observation that the maturation of yeast Grx3/4 and human GLRX3-BOLA2 heterodimers requires the mitochondrial ISC machinery but not CIA components (Mühlenhoff et al., 2010; Frey et al., 2016), it is concluded that cytosolic class II GRXs should build their cluster from a sulfur compound exported by the mitochondrial ATM transporter (Figure 1C). In yeast *grx3/4*Δ, the Fe or Fe-cofactor insertion in various proteins present in cytosol [catalase, ribonucleotide reductase (RNR)], and mitochondria (complexes II and III, aconitase, Coq7 mono-oxygenase) is altered (Mühlenhoff et al., 2010; Zhang et al., 2011). Moreover, the respective increased and decreased Fe levels in cytosol and mitochondria of Grx3/4 depleted cells pointed to impaired Fe distribution (Mühlenhoff et al., 2010). These additional functions of yeast Grx3/4 are well exemplified in the case of RNR di-iron cofactor biogenesis because Grx4 provides the Fe atoms, but also serves for the maturation of holo-Dre2, that provides the required electrons (Li et al., 2017). A contribution of yeast Bol2 for these functions is unclear even though a general role in cytosolic Fe–S protein maturation is excluded (Uzarska et al., 2016). In human, GLRX3-BOLA2 trimeric complexes bridging two [2Fe–2S] clusters were proposed to constitute a reservoir for delivering Fe or Fe–S cluster to some Fe-containing target proteins based notably on the six–eightfold increased abundance observed in response to elevated iron (Frey et al., 2016).

The function of GRXS17, the sole nucleo-cytosolic class II GRX in plants, has been explored using several approaches. Tandem affinity purification using a tagged GRX form expressed in Arabidopsis cell cultures and seedlings pointed to the association of GRXS17 with CIA components and BOLA2 (Iñigo et al., 2016). The interactions with DRE2 and BOLA2 have been confirmed *in vivo* by binary yeast two-hybrid and BiFC and/or *in vitro* by co-expression in *E. coli* (Couturier et al., 2014; Dhalleine et al., 2014; Iñigo et al., 2016). As GRXs interact with Dre2/Anamorsin in yeast and human cells (Zhang et al., 2011; Banci et al., 2015b), the only direct CIA partner of GRXS17 might be DRE2 and the other proteins part of a complex. Besides, the binding of GRXS17 with putative Fe–S client proteins involved in purine salvage (xanthine dehydrogenase 1) or tRNA modification (thiouridylase subunits 1 and 2) was shown (Iñigo et al., 2016). Thus, one would expect that plants deficient in GRXS17 display a marked phenotype in relation with Fe metabolism, but the analysis of Arabidopsis *grxs17* plants led to relatively complex data. Indeed, their development is only mildly affected under standard growth conditions, but gets severely impaired (elongated leaves, modified shoot apical meristem structure, and altered auxin response) at high temperature or under long photoperiod (Cheng et al., 2011; Knuesting et al., 2015). It is not yet clear whether a redox- and/or an Fe-related function of GRXS17 is responsible for these alterations. In fact, *in vitro* pull-downs performed using the recombinant protein allowed recovering many non-Fe–S proteins including the NF-YC11 transcriptional regulator (Knuesting et al., 2015). Moreover, there is no variation in the Fe content in mature leaves and only a slight increase in seeds (Yu et al., 2017) of *grxs17* plants that exhibit no or minor decreases in the activity of three Fe–S containing enzymes: aconitase, aldehyde oxidase and xanthine dehydrogenase (Knuesting et al., 2015; Iñigo et al., 2016). On the other hand, GRXS17-deficient lines exhibit a slightly increased sensitivity to genotoxic stress which is reminiscent of mutants compromised in the CIA pathway (Iñigo et al., 2016). Finally, when GRXS17-deficient lines are exposed to Fe deficiency, the primary root growth reduction, that is already visible under standard conditions, is exacerbated and ROS levels are elevated (Iñigo et al., 2016; Yu et al., 2017). Whether plant GRXS17 and BOLA2 act in concert remains unclear. The Arabidopsis *bola2* (incorrectly named *bola3*) mutant displays no phenotype under control conditions and no change in the activity of typical Fe–S enzymes (Qin et al., 2015). Surprisingly, this line is more tolerant to oxidative stress generated by an Fe excess (Qin et al., 2015). In conclusion, *bola2* and *grxs17* plants exhibit relatively mild phenotypes, visible mostly under stress conditions, compared to those described for human and yeast orthologs and to the embryo-lethality of most Arabidopsis mutants defective for early acting CIA components (Bernard et al., 2013). This raises some questions about the exact functions of BOLA2 and GRXS17 in the regulation of Fe homeostasis in plant cells and about the existence of an alternative system, notably for delivering Fe–S clusters to DRE2, whose function is essential.

## Roadmap Toward the Understanding of the Roles of GRX/Bola Couples in Plants

In this section, we propose some ideas and experimental strategies that should warrant deciphering the functions associated to GRX/BOLA couples in plants.

Evidence obtained so far indicate that the class II mono-domain GRXs and BOLAs present in mitochondria of non-plant eukaryotes and in bacteria act as maturation factors for the biogenesis of Fe–S proteins. A similar role seems true for the plant mitochondrial GRXS15, but it is now mandatory to examine whether it also contributes to the maturation of extra-mitochondrial proteins. Another challenge will be to understand why it is essential in plants unlike in yeast. Also, the physiological consequences of *BOLA4* depletion must be investigated to see whether this fits with a function connected to GRXS15. Concerning plastidial proteins (GRXS14, GRXS16, BOLA1, and BOLA4), a role in the maturation of Fe–S proteins still needs to be demonstrated *in planta*, despite they can functionally substitute to their mitochondrial yeast counterparts.

With regard to the cytosolic multi-domain GRXs and BOLAs, a role in Fe metabolism seems evolutionary conserved, but their contribution and partners differ. In yeast, their primary function is to regulate Fe-responsive transcription factors. Additional functions are to ensure a proper Fe distribution toward all types of Fe cofactors (including heme and non-heme Fe centers) and/or to serve for Dre2 maturation, thus contributing to the correct functioning of the CIA machinery. In this case, Grx3/4 have an exclusive or predominant role because the corresponding mutant is lethal or strongly affected, unlike the *bol2/fra2* mutant. Experimental evidence indicate that the involvement of GRX and/or BOLA in DRE2 maturation is likely also true in mammals and plants, but evidence supporting other functions are scarce.

A first prerequisite to future molecular and physiological analyses is to generate the missing single knock-out lines but also multiple knock-out lines for possibly redundant proteins. This would be particularly important to obtain lines combining mutations for GRXS14 and GRXS16, for BOLA1 and BOLA4, but also for GRXS17 and the only other Fe–S ligating GRXs reported so far in the cytosol, namely GRXC1 (Rouhier et al., 2007), or BOLA2. In case the single or multiple mutants are lethal, an option for obtaining viable lines would be to generate RNAi lines as for *GRXS15*, but also dominant negative mutant lines expressing mutated versions of GRX or BOLA unable for instance to ligate the Fe–S cluster, i.e., mutated for the catalytic cysteine of GRXs or the conserved histidine residue of BOLA.

At the physiological level, the growth of these plants should be analyzed under standard conditions, but also under environmental constraints as the shoot phenotypes of *grxs17* mutants are only visible in specific conditions. For the BOLA2-GRXS17 couple, understanding their connection and discriminating between Fe- or redox-related functions will require in particular to assess the phenotypes of the corresponding mutants in the same experimental setup and conditions. Considering the described importance of GSH for ligating Fe–S cluster in GRX homodimer or GRX-BOLA heterodimer and for the maturation of cytosolic Fe–S proteins (Sipos et al., 2002), crossing some of these mutants with mutants having an altered GSH homeostasis would certainly be informative.

In other respects, an obvious strategy is to measure the abundance/activity of representative Fe–S proteins in these lines. However, performing quantitative proteomic and metabolomic approaches may be more informative and help obtaining a broader view of the molecular and cellular mechanisms affected and of the compensations established. It may also rapidly point to metabolic differences existing among mutants.

In all cases, determining the identity of the direct and indirect targets of both GRXs and BOLAs would represent a mandatory information. For instance, the proteins involved in the Fe–S cluster maturation process may act at different steps. Various approaches complementary to quantitative proteomics proved valuable even for detecting supposedly transient interactions among Fe–S cluster donors and acceptors (Touraine et al., 2019). Hence, it is possible to combine it to another non-targeted approach such as co-immunoprecipitation or to binary yeast two-hybrid experiments which has the advantage for instance to allow studying rapidly sequence requirements by mutational analysis.

In summary, the combination of genetic approaches, omics analyses and conventional biochemical tools should in principle allow better delineating the roles and specificities of GRX/BOLA couples in the maintenance of Fe homeostasis in plants.

## Data Availability

All datasets analyzed for this study are included in the manuscript and the Supplementary Files.

## Author Contributions

All authors wrote the text and approved the final version of the manuscript.

## Conflict of Interest Statement

The authors declare that the research was conducted in the absence of any commercial or financial relationships that could be construed as a potential conflict of interest.

## References

[B1] Aldea M. Garrido T. Hernández-Chico C. Vicente M. Kushner S. R. (1989). Induction of a growth-phase-dependent promoter triggers transcription of bolA, an *Escherichia coli* morphogene. *EMBO J.* 8 3923–3931. 10.1002/j.1460-2075.1989.tb08573.x 2684651 PMC402084

[B2] Alves R. Vilaprinyo E. Sorribas A. Herrero E. (2009). Evolution based on domain combinations: the case of glutaredoxins. *BMC Evol. Biol.* 9:66. 10.1186/1471-2148-9-66 19321008 PMC2679010

[B3] Attarian R. Hu G. Sánchez-León E. Caza M. Croll D. Do E. (2018). The monothiol glutaredoxin grx4 regulates iron homeostasis and virulence in *Cryptococcus neoformans*. *mBio* 9:e02377-18. 10.1128/mBio.02377-18 30514787 PMC6282196

[B4] Banci L. Camponeschi F. Ciofi-Baffoni S. Muzzioli R. (2015a). Elucidating the molecular function of human BOLA2 in GRX3-dependent anamorsin maturation pathway. *J. Am. Chem. Soc.* 137 16133–16143. 10.1021/jacs.5b10592 26613676

[B5] Banci L. Ciofi-Baffoni S. Gajda K. Muzzioli R. Peruzzini R. Winkelmann J. (2015b). N-terminal domains mediate [2Fe-2S] cluster transfer from glutaredoxin-3 to anamorsin. *Nat. Chem. Biol.* 11 772–778. 10.1038/nchembio.1892 26302480

[B6] Bandyopadhyay S. Gama F. Molina-Navarro M. M. Gualberto J. M. Claxton R. Naik S. G. (2008). Chloroplast monothiol glutaredoxins as scaffold proteins for the assembly and delivery of [2Fe-2S] clusters. *EMBO J.* 27 1122–1133. 10.1038/emboj.2008.50 18354500 PMC2323258

[B7] Barupala D. P. Dzul S. P. Riggs-Gelasco P. J. Stemmler T. L. (2016). Synthesis, delivery and regulation of eukaryotic heme and Fe-S cluster cofactors. *Arch. Biochem. Biophys.* 592 60–75. 10.1016/j.abb.2016.01.010 26785297 PMC4784227

[B8] Benyamina S. M. Baldacci-Cresp F. Couturier J. Chibani K. Hopkins J. Bekki A. (2013). Two *Sinorhizobium meliloti* glutaredoxins regulate iron metabolism and symbiotic bacteroid differentiation. *Environ. Microbiol.* 15 795–810. 10.1111/j.1462-2920.2012.02835.x 22891731

[B9] Bernard D. G. Netz D. J. A. Lagny T. J. Pierik A. J. Balk J. (2013). Requirements of the cytosolic iron-sulfur cluster assembly pathway in *Arabidopsis*. *Philos. Trans. R. Soc. Lond. B. Biol. Sci.* 368:20120259. 10.1098/rstb.2012.0259 23754812 PMC3685461

[B10] Boutigny S. Saini A. Baidoo E. E. K. Yeung N. Keasling J. D. Butland G. (2013). Physical and functional interactions of a monothiol glutaredoxin and an iron sulfur cluster carrier protein with the sulfur-donating radical S-adenosyl-L-methionine enzyme MiaB. *J. Biol. Chem.* 288 14200–14211. 10.1074/jbc.M113.460360 23543739 PMC3656276

[B11] Brault A. Mourer T. Labbé S. (2015). Molecular basis of the regulation of iron homeostasis in fission and filamentous yeasts. *IUBMB Life* 67 801–815. 10.1002/iub.1441 26472434

[B12] Burschel S. Kreuzer Decovic D. Nuber F. Stiller M. Hofmann M. Zupok A. (2019). Iron-sulfur cluster carrier proteins involved in the assembly of *Escherichia coli* NADH:ubiquinone oxidoreductase (complex I). *Mol. Microbiol.* 111 31–45. 10.1111/mmi.14137 30251413

[B13] Butland G. Babu M. Díaz-Mejía J. J. Bohdana F. Phanse S. Gold B. (2008). eSGA: *E. coli* synthetic genetic array analysis. *Nat. Methods* 5 789–795. 10.1038/nmeth.1239 18677321

[B14] Camaschella C. Campanella A. De Falco L. Boschetto L. Merlini R. Silvestri L. (2007). The human counterpart of zebrafish shiraz shows sideroblastic-like microcytic anemia and iron overload. *Blood* 110 1353–1358. 10.1182/blood-2007-02-072520 17485548

[B15] Cameron J. M. Janer A. Levandovskiy V. Mackay N. Rouault T. A. Tong W.-H. (2011). Mutations in iron-sulfur cluster scaffold genes NFU1 and BOLA3 cause a fatal deficiency of multiple respiratory chain and 2-oxoacid dehydrogenase enzymes. *Am. J. Hum. Genet.* 89 486–495. 10.1016/j.ajhg.2011.08.011 21944046 PMC3188835

[B16] Cheng N.-H. Liu J.-Z. Liu X. Wu Q. Thompson S. M. Lin J. (2011). *Arabidopsis* monothiol glutaredoxin, AtGRXS17, is critical for temperature-dependent postembryonic growth and development via modulating auxin response. *J. Biol. Chem.* 286 20398–20406. 10.1074/jbc.M110.201707 21515673 PMC3121514

[B17] Chung W.-H. Kim K.-D. Roe J.-H. (2005). Localization and function of three monothiol glutaredoxins in *Schizosaccharomyces pombe*. *Biochem. Biophys. Res. Commun.* 330 604–610. 10.1016/j.bbrc.2005.02.183 15796926

[B18] Comini M. A. Rettig J. Dirdjaja N. Hanschmann E.-M. Berndt C. Krauth-Siegel R. L. (2008). Monothiol glutaredoxin-1 is an essential iron-sulfur protein in the mitochondrion of African trypanosomes. *J. Biol. Chem.* 283 27785–27798. 10.1074/jbc.M802010200 18669638

[B19] Couturier J. Jacquot J.-P. Rouhier N. (2009). Evolution and diversity of glutaredoxins in photosynthetic organisms. *Cell. Mol. Life Sci.* 66 2539–2557. 10.1007/s00018-009-0054-y 19506802 PMC11115520

[B20] Couturier J. Przybyla-Toscano J. Roret T. Didierjean C. Rouhier N. (2015). The roles of glutaredoxins ligating Fe-S clusters: sensing, transfer or repair functions? *Biochim. Biophys. Acta* 1853 1513–1527. 10.1016/j.bbamcr.2014.09.018 25264274

[B21] Couturier J. Touraine B. Briat J.-F. Gaymard F. Rouhier N. (2013). The iron-sulfur cluster assembly machineries in plants: current knowledge and open questions. *Front. Plant Sci.* 4:259. 10.3389/fpls.2013.00259 23898337 PMC3721309

[B22] Couturier J. Wu H.-C. Dhalleine T. Pégeot H. Sudre D. Gualberto J. M. (2014). Monothiol glutaredoxin-BolA interactions: redox control of *Arabidopsis thaliana* BolA2 and SufE1. *Mol. Plant* 7 187–205. 10.1093/mp/sst156 24203231

[B23] Dhalleine T. Rouhier N. Couturier J. (2014). Putative roles of glutaredoxin-BolA holo-heterodimers in plants. *Plant Signal. Behav.* 9:e28564. 10.4161/psb.28564 24714563 PMC4091568

[B24] Dlouhy A. C. Beaudoin J. Labbé S. Outten C. E. (2017). *Schizosaccharomyces pombe* Grx4 regulates the transcriptional repressor Php4 via [2Fe-2S] cluster binding. *Met. Integr. Biometal Sci.* 9 1096–1105. 10.1039/c7mt00144d 28725905 PMC5595146

[B25] Dlouhy A. C. Li H. Albetel A.-N. Zhang B. Mapolelo D. T. Randeniya S. (2016). The *Escherichia coli* BolA protein IbaG forms a histidine-Ligated [2Fe-2S]-bridged complex with Grx4. *Biochemistry* 55 6869–6879. 10.1021/acs.biochem.6b00812 27951647 PMC5258196

[B26] Encinar del Dedo J. Gabrielli N. Carmona M. Ayté J. Hidalgo E. (2015). A cascade of iron-containing proteins governs the genetic iron starvation response to promote iron uptake and inhibit iron storage in fission yeast. *PLoS Genet.* 11:e1005106. 10.1371/journal.pgen.1005106 25806539 PMC4373815

[B27] Frey A. G. Palenchar D. J. Wildemann J. D. Philpott C. C. (2016). A glutaredoxin ⋅ bola complex serves as an iron-sulfur cluster chaperone for the cytosolic cluster assembly machinery. *J. Biol. Chem.* 291 22344–22356. 10.1074/jbc.M116.744946 27519415 PMC5077177

[B28] Guo B. Phillips J. D. Yu Y. Leibold E. A. (1995). Iron regulates the intracellular degradation of iron regulatory protein 2 by the proteasome. *J. Biol. Chem.* 270 21645–21651. 10.1074/jbc.270.37.21645 7665579

[B29] Haunhorst P. Hanschmann E.-M. Bräutigam L. Stehling O. Hoffmann B. Mühlenhoff U. (2013). Crucial function of vertebrate glutaredoxin 3 (PICOT) in iron homeostasis and hemoglobin maturation. *Mol. Biol. Cell* 24 1895–1903. 10.1091/mbc.E12-09-0648 23615448 PMC3681695

[B30] Iñigo S. Durand A. N. Ritter A. Le Gall S. Termathe M. Klassen R. (2016). Glutaredoxin GRXS17 associates with the cytosolic iron-sulfur cluster assembly pathway. *Plant Physiol.* 172 858–873. 10.1104/pp.16.00261 27503603 PMC5047072

[B31] Jacques J.-F. Mercier A. Brault A. Mourer T. Labbé S. (2014). Fra2 is a co-regulator of Fep1 inhibition in response to iron starvation. *PLoS One* 9:e98959. 10.1371/journal.pone.0098959 24897379 PMC4045890

[B32] Kim K.-D. Chung W.-H. Kim H.-J. Lee K.-C. Roe J.-H. (2010). Monothiol glutaredoxin Grx5 interacts with Fe-S scaffold proteins Isa1 and Isa2 and supports Fe-S assembly and DNA integrity in mitochondria of fission yeast. *Biochem. Biophys. Res. Commun.* 392 467–472. 10.1016/j.bbrc.2010.01.051 20085751

[B33] Knuesting J. Riondet C. Maria C. Kruse I. Bécuwe N. König N. (2015). Arabidopsis glutaredoxin S17 and its partner, the nuclear factor Y subunit C11/negative cofactor 2α, contribute to maintenance of the shoot apical meristem under long-day photoperiod. *Plant Physiol.* 167 1643–1658. 10.1104/pp.15.00049 25699589 PMC4378178

[B34] Kumánovics A. Chen O. S. Li L. Bagley D. Adkins E. M. Lin H. (2008). Identification of FRA1 and FRA2 as genes involved in regulating the yeast iron regulon in response to decreased mitochondrial iron-sulfur cluster synthesis. *J. Biol. Chem.* 283 10276–10286. 10.1074/jbc.M801160200 18281282 PMC2447656

[B35] Lesuisse E. Knight S. A. B. Courel M. Santos R. Camadro J.-M. Dancis A. (2005). Genome-wide screen for genes with effects on distinct iron uptake activities in *Saccharomyces cerevisiae*. *Genetics* 169 107–122. 10.1534/genetics.104.035873 15489514 PMC1448889

[B36] Li H. Mapolelo D. T. Dingra N. N. Keller G. Riggs-Gelasco P. J. Winge D. R. (2011). Histidine 103 in Fra2 is an iron-sulfur cluster ligand in the [2Fe-2S] Fra2-Grx3 complex and is required for *in vivo* iron signaling in yeast. *J. Biol. Chem.* 286 867–876. 10.1074/jbc.M110.184176 20978135 PMC3013046

[B37] Li H. Mapolelo D. T. Randeniya S. Johnson M. K. Outten C. E. (2012). Human glutaredoxin 3 forms [2Fe-2S]-bridged complexes with human BolA2. *Biochemistry* 51 1687–1696. 10.1021/bi2019089 22309771 PMC3331715

[B38] Li H. Outten C. E. (2012). Monothiol CGFS glutaredoxins and BolA-like proteins: [2Fe-2S] binding partners in iron homeostasis. *Biochemistry* 51 4377–4389. 10.1021/bi300393z 22583368 PMC3448021

[B39] Li H. Stümpfig M. Zhang C. An X. Stubbe J. Lill R. (2017). The diferric-tyrosyl radical cluster of ribonucleotide reductase and cytosolic iron-sulfur clusters have distinct and similar biogenesis requirements. *J. Biol. Chem.* 292 11445–11451. 10.1074/jbc.M117.786178 28515324 PMC5500809

[B40] Lill R. (2009). Function and biogenesis of iron-sulphur proteins. *Nature* 460 831–838. 10.1038/nature08301 19675643

[B41] Mapolelo D. T. Zhang B. Randeniya S. Albetel A.-N. Li H. Couturier J. (2013). Monothiol glutaredoxins and A-type proteins: partners in Fe-S cluster trafficking. *Dalton Trans. Camb. Engl.* 2003 3107–3115. 10.1039/c2dt32263c 23292141 PMC3578507

[B42] Melber A. Na U. Vashisht A. Weiler B. D. Lill R. Wohlschlegel J. A. (2016). Role of Nfu1 and Bol3 in iron-sulfur cluster transfer to mitochondrial clients. *eLife* 5:e15991. 10.7554/eLife.15991 27532773 PMC5014551

[B43] Mil-Homens D. Barahona S. Moreira R. N. Silva I. J. Pinto S. N. Fialho A. M. (2018). Stress response protein bola influences fitness and promotes *Salmonella enterica Serovar Typhimurium* virulence. *Appl. Environ. Microbiol.* 84:e02850-17. 10.1128/AEM.02850-17 29439986 PMC5881071

[B44] Molina M. M. Bellí G. de la Torre M. A. Rodríguez-Manzaneque M. T. Herrero E. (2004). Nuclear monothiol glutaredoxins of *Saccharomyces cerevisiae* can function as mitochondrial glutaredoxins. *J. Biol. Chem.* 279 51923–51930. 10.1074/jbc.M410219200 15456753

[B45] Moseler A. Aller I. Wagner S. Nietzel T. Przybyla-Toscano J. Mühlenhoff U. (2015). The mitochondrial monothiol glutaredoxin S15 is essential for iron-sulfur protein maturation in *Arabidopsis thaliana*. *Proc. Natl. Acad. Sci. U.S.A.* 112 13735–13740. 10.1073/pnas.1510835112 26483494 PMC4640787

[B46] Mühlenhoff U. Gerber J. Richhardt N. Lill R. (2003). Components involved in assembly and dislocation of iron-sulfur clusters on the scaffold protein Isu1p. *EMBO J.* 22 4815–4825. 10.1093/emboj/cdg446 12970193 PMC212715

[B47] Mühlenhoff U. Molik S. Godoy J. R. Uzarska M. A. Richter N. Seubert A. (2010). Cytosolic monothiol glutaredoxins function in intracellular iron sensing and trafficking via their bound iron-sulfur cluster. *Cell Metab.* 12 373–385. 10.1016/j.cmet.2010.08.001 20889129 PMC4714545

[B48] Nasta V. Giachetti A. Ciofi-Baffoni S. Banci L. (2017). Structural insights into the molecular function of human [2Fe-2S] BOLA1-GRX5 and [2Fe-2S] BOLA3-GRX5 complexes. *Biochim. Biophys. Acta* 1861 2119–2131. 10.1016/j.bbagen.2017.05.005 28483642

[B49] Ojeda L. Keller G. Muhlenhoff U. Rutherford J. C. Lill R. Winge D. R. (2006). Role of glutaredoxin-3 and glutaredoxin-4 in the iron regulation of the Aft1 transcriptional activator in *Saccharomyces cerevisia*e. *J. Biol. Chem.* 281 17661–17669. 10.1074/jbc.M602165200 16648636

[B50] Outten C. E. Albetel A.-N. (2013). Iron sensing and regulation in *Saccharomyces cerevisiae*: ironing out the mechanistic details. *Curr. Opin. Microbiol.* 16 662–668. 10.1016/j.mib.2013.07.020 23962819 PMC3842356

[B51] Philpott C. C. Ryu M.-S. Frey A. Patel S. (2017). Cytosolic iron chaperones: proteins delivering iron cofactors in the cytosol of mammalian cells. *J. Biol. Chem.* 292 12764–12771. 10.1074/jbc.R117.791962 28615454 PMC5546017

[B52] Poor C. B. Wegner S. V. Li H. Dlouhy A. C. Schuermann J. P. Sanishvili R. (2014). Molecular mechanism and structure of the *Saccharomyces cerevisiae* iron regulator Aft2. *Proc. Natl. Acad. Sci. U.S.A.* 111 4043–4048. 10.1073/pnas.1318869111 24591629 PMC3964038

[B53] Przybyla-Toscano J. Roret T. Couturier J. Rouhier N. (2017). “FeS Cluster Assembly: Role of Monothiol Grxs and Nfu Proteins,” in *Encyclopedia of Inorganic and Bioinorganic Chemistry*, eds Scott R. A (New York, NY: American Cancer Society), 1–19. 10.1002/9781119951438.eibc2470

[B54] Pujol-Carrion N. Belli G. Herrero E. Nogues A. de la Torre-Ruiz M. A. (2006). Glutaredoxins Grx3 and Grx4 regulate nuclear localisation of Aft1 and the oxidative stress response in *Saccharomyces cerevisiae*. *J. Cell Sci.* 119 4554–4564. 10.1242/jcs.03229 17074835

[B55] Qin L. Wang M. Zuo J. Feng X. Liang X. Wu Z. (2015). Cytosolic bolA plays a repressive role in the tolerance against excess iron and mv-induced oxidative stress in plants. *PLoS One* 10:e0124887. 10.1371/journal.pone.0124887 25928219 PMC4415784

[B56] Rey P. Becuwe N. Tourrette S. Rouhier N. (2017). Involvement of *Arabidopsis* glutaredoxin S14 in the maintenance of chlorophyll content. *Plant Cell Environ.* 40 2319–2332. 10.1111/pce.13036 28741719

[B57] Rietzschel N. Pierik A. J. Bill E. Lill R. Mühlenhoff U. (2015). The basic leucine zipper stress response regulator Yap5 senses high-iron conditions by coordination of [2Fe-2S] clusters. *Mol. Cell. Biol.* 35 370–378. 10.1128/MCB.01033-14 25368382 PMC4272429

[B58] Rodríguez-Manzaneque M. T. Tamarit J. Bellí G. Ros J. Herrero E. (2002). Grx5 is a mitochondrial glutaredoxin required for the activity of iron/sulfur enzymes. *Mol. Biol. Cell* 13 1109–1121. 10.1091/mbc.01-10-0517 11950925 PMC102255

[B59] Roret T. Tsan P. Couturier J. Zhang B. Johnson M. K. Rouhier N. (2014). Structural and spectroscopic insights into BolA-glutaredoxin complexes. *J. Biol. Chem.* 289 24588–24598. 10.1074/jbc.M114.572701 25012657 PMC4148882

[B60] Rouault T. A. Maio N. (2017). Biogenesis and functions of mammalian iron-sulfur proteins in the regulation of iron homeostasis and pivotal metabolic pathways. *J. Biol. Chem.* 292 12744–12753. 10.1074/jbc.R117.789537 28615439 PMC5546015

[B61] Rouhier N. Unno H. Bandyopadhyay S. Masip L. Kim S.-K. Hirasawa M. (2007). Functional, structural, and spectroscopic characterization of a glutathione-ligated [2Fe-2S] cluster in poplar glutaredoxin C1. *Proc. Natl. Acad. Sci. U.S.A.* 104 7379–7384. 10.1073/pnas.0702268104 17460036 PMC1863468

[B62] Sipos K. Lange H. Fekete Z. Ullmann P. Lill R. Kispal G. (2002). Maturation of cytosolic iron-sulfur proteins requires glutathione. *J. Biol. Chem.* 277 26944–26949. 10.1074/jbc.M200677200 12011041

[B63] Ströher E. Grassl J. Carrie C. Fenske R. Whelan J. Millar A. H. (2016). Glutaredoxin S15 is involved in Fe-S cluster transfer in mitochondria influencing lipoic acid-dependent enzymes, plant growth, and arsenic tolerance in *Arabidopsis*. *Plant Physiol.* 170 1284–1299. 10.1104/pp.15.01308 26672074 PMC4775112

[B64] Touraine B. Vignols F. Przybyla-Toscano J. Ischebeck T. Dhalleine T. Wu H.-C. (2019). Iron-sulfur protein NFU2 is required for branched-chain amino acid synthesis in Arabidopsis roots. *J. Exp. Bot.* 70 1875–1889. 10.1093/jxb/erz050 30785184

[B65] Ueta R. Fujiwara N. Iwai K. Yamaguchi-Iwai Y. (2012). Iron-induced dissociation of the Aft1p transcriptional regulator from target gene promoters is an initial event in iron-dependent gene suppression. *Mol. Cell. Biol.* 32 4998–5008. 10.1128/MCB.00726-12 23045394 PMC3510542

[B66] Uzarska M. A. Dutkiewicz R. Freibert S.-A. Lill R. Mühlenhoff U. (2013). The mitochondrial Hsp70 chaperone Ssq1 facilitates Fe/S cluster transfer from Isu1 to Grx5 by complex formation. *Mol. Biol. Cell* 24 1830–1841. 10.1091/mbc.E12-09-0644 23615440 PMC3681689

[B67] Uzarska M. A. Nasta V. Weiler B. D. Spantgar F. Ciofi-Baffoni S. Saviello M. R. (2016). Mitochondrial Bol1 and Bol3 function as assembly factors for specific iron-sulfur proteins. *eLife* 5:e16673. 10.7554/eLife.16673 27532772 PMC5014550

[B68] Uzarska M. A. Przybyla-Toscano J. Spantgar F. Zannini F. Lill R. Mühlenhoff U. (2018). Conserved functions of Arabidopsis mitochondrial late-acting maturation factors in the trafficking of iron-sulfur clusters. *Biochim. Biophys. Acta Mol. Cell Res.* 1865 1250–1259. 10.1016/j.bbamcr.2018.06.003 29902489

[B69] Willems P. Wanschers B. F. J. Esseling J. Szklarczyk R. Kudla U. Duarte I. (2013). BOLA1 is an aerobic protein that prevents mitochondrial morphology changes induced by glutathione depletion. *Antioxid. Redox Signal.* 18 129–138. 10.1089/ars.2011.4253 22746225 PMC3513987

[B70] Wingert R. A. Galloway J. L. Barut B. Foott H. Fraenkel P. Axe J. L. (2005). Deficiency of glutaredoxin 5 reveals Fe-S clusters are required for vertebrate haem synthesis. *Nature* 436 1035–1039. 10.1038/nature03887 16110529

[B71] Xia H. Li B. Zhang Z. Wang Q. Qiao T. Li K. (2015). Human glutaredoxin 3 can bind and effectively transfer [4Fe-4S] cluster to apo-iron regulatory protein 1. *Biochem. Biophys. Res. Commun.* 465 620–624. 10.1016/j.bbrc.2015.08.073 26296460

[B72] Ye H. Jeong S. Y. Ghosh M. C. Kovtunovych G. Silvestri L. Ortillo D. (2010). Glutaredoxin 5 deficiency causes sideroblastic anemia by specifically impairing heme biosynthesis and depleting cytosolic iron in human erythroblasts. *J. Clin. Invest.* 120 1749–1761. 10.1172/JCI40372 20364084 PMC2860907

[B73] Yeung N. Gold B. Liu N. L. Prathapam R. Sterling H. J. Willams E. R. (2011). The *E. coli* monothiol glutaredoxin GrxD forms homodimeric and heterodimeric FeS cluster containing complexes. *Biochemistry* 50 8957–8969. 10.1021/bi2008883 21899261 PMC3236052

[B74] Yu H. Yang J. Shi Y. Donelson J. Thompson S. M. Sprague S. (2017). *Arabidopsis* glutaredoxin S17 contributes to vegetative growth, mineral accumulation, and redox balance during iron deficiency. *Front. Plant Sci.* 8:1045. 10.3389/fpls.2017.01045 28674546 PMC5474874

[B75] Zhang Y. Liu L. Wu X. An X. Stubbe J. Huang M. (2011). Investigation of in vivo diferric tyrosyl radical formation in *Saccharomyces cerevisiae* Rnr2 protein: requirement of Rnr4 and contribution of Grx3/4 AND Dre2 proteins. *J. Biol. Chem.* 286 41499–41509. 10.1074/jbc.M111.294074 21931161 PMC3308861

